# Resmetirom: A Breakthrough in the Treatment of Metabolic Dysfunction–Associated Steatotic Liver Disease (MASLD)

**DOI:** 10.1002/hsr2.70920

**Published:** 2025-06-18

**Authors:** Laiba Shakeel, Ayesha Shaukat, Aymar Akilimali

**Affiliations:** ^1^ Department of Internal Medicine Dow University of Health Sciences Karachi Pakistan; ^2^ Department of Research Medical Research Circle (MedReC) Goma Democratic Republic of the Congo

**Keywords:** cardiometabolic risk factors, Diabetes Mellitus Type 2, elasticity imaging techniques, MASLD, metabolic syndrome, non‐alcoholic fatty liver disease, Resmetirom, thyroid hormone receptors beta

## Abstract

**Background and Aims:**

Metabolic dysfunction–associated steatotic liver disease (MASLD), previously referred to as non‐alcoholic fatty liver disease (NAFLD), has become the most prevalent chronic liver disease worldwide. Its increasing incidence is closely linked with rising rates of obesity, Type 2 diabetes mellitus (T2DM), and metabolic syndrome. MASLD includes a spectrum of liver pathologies such as fibrosis, cirrhosis, and metabolic dysfunction–associated steatohepatitis (MASH). This review explores diagnostic advancements and therapeutic developments, focusing on Resmetirom, a selective thyroid hormone receptor‐beta (THR‐β) agonist, as a novel pharmacologic intervention.

**Methods:**

The review draws upon current literature, clinical guidelines, and data from pivotal randomized controlled trials to evaluate the diagnostic criteria, non‐invasive assessment tools, lifestyle recommendations, and emerging pharmacotherapy for MASLD and MASH, focusing on Resmetirom.

**Results:**

Resmetirom has demonstrated significant efficacy in reducing hepatic fat content and improving markers of liver fibrosis in patients with noncirrhotic MASH and fibrosis stages F2–F3. Clinical trials showed improved liver histology, lipid profiles, and MASH resolution without fibrosis progression. Despite its promising profile, gastrointestinal side effects and gallstone‐related complications have been observed. Long‐term safety and outcomes related to cirrhosis or hepatocellular carcinoma prevention remain under investigation.

**Conclusion:**

MASLD is a significant public health concern driven by metabolic dysfunction. While lifestyle changes remain central to management, Resmetirom offers a promising pharmacological option for patients with moderate to advanced MASH. Further long‐term studies are needed to fully establish its safety, effectiveness, and role in preventing advanced liver disease.

## 1 Introduction

Metabolic dysfunction–associated steatotic liver disease (MASLD), previously known as non‐alcoholic fatty liver disease (NAFLD), is a significant global health problem characterized by macrovesicular steatosis involving at least 5% of hepatocytes without secondary causes such as alcohol or drug abuse [1]. MASLD encompasses a spectrum of liver conditions, including cirrhosis, fibrosis, and what was formerly termed non‐alcoholic steatohepatitis (NASH), now recognized as metabolic dysfunction–associated steatohepatitis (MASH) [1].

MASLD has become the leading cause of chronic liver disease worldwide, with its prevalence steadily rising. Current data indicate that approximately 25% of the global population is affected by hepatic steatosis, with higher rates observed in individuals with obesity (BMI ≥ 30 kg/m²), Type 2 diabetes mellitus (T2DM), and those who have undergone bariatric surgery [2, 3]. Trend analysis shows a sharp increase in MASLD prevalence, from 25.3% during 1990–2006 to 38.2% during 2016–2019, marking a 50.4% increase over nearly three decades [4]. In 2019, it was estimated that 1.66 billion adults aged 20 and above globally were living with MASLD, with the highest prevalence recorded in East Asia [4].

Gender disparities persist in disease prevalence; men are more likely to develop MASH, whereas postmenopausal women exhibit a higher overall prevalence of MASLD [4, 5]. The disease poses significant health risks, including the progression to cirrhosis, hepatocellular carcinoma (HCC), the need for liver transplantation, and increased mortality due to cardiovascular diseases [4, 5].

The updated diagnostic criteria for MASLD require confirmed hepatic steatosis through imaging or biopsy alongside at least one cardiometabolic risk factor (CMRF) [6]. These risk factors include being overweight or obese (BMI ≥ 25 kg/m², or ≥ 23 kg/m² for Asian populations), having T2DM, hypertension (blood pressure ≥ 130/85 mmHg or use of antihypertensive medication), hypertriglyceridemia (plasma triglycerides ≥ 150 mg/dL or ongoing lipid‐lowering therapy), and low HDL cholesterol (≤ 40 mg/dL in men and ≤ 50 mg/dL in women) [6, 7, 8].

Furthermore, a new classification known as Metabolic and Alcohol‐Related Liver Disease (MetALD) has been introduced to categorize individuals with MASLD who consume moderate‐to‐high amounts of alcohol (140–350 g/week for females and 210–420 g/week for males), recognizing the complex relationship between metabolic dysfunction and alcohol intake [8, 9]. This evolution in terminology and diagnostic criteria aims to understand the disease spectrum better, reduce the stigma associated with liver disease, and improve classification for better clinical management and research.

## 2 Diagnosis

Patients with elevated liver transaminases or gamma‐glutamyltransferase, especially in the presence of hepatic steatosis on abdominal ultrasonography and features of metabolic syndrome, should raise clinical suspicion for MASLD [10]. Although imaging modalities effectively detect hepatic fat accumulation, they cannot differentiate MASH from simple steatosis [10]. Therefore, liver biopsy remains the gold standard for confirming MASH and assessing key histological features such as inflammation and fibrosis [1].

Given the invasive nature, cost, and procedural risks associated with liver biopsy, there has been a growing emphasis on non‐invasive diagnostic tools for assessing and monitoring MASLD. Among imaging modalities, transient elastography (FibroScan) is widely used in clinical practice to evaluate liver stiffness and steatosis via a controlled attenuation parameter (CAP), offering a rapid, reproducible, and painless alternative [11, 12]. Magnetic resonance imaging–proton density fat fraction (MRI‐PDFF) provides a more precise quantification of hepatic fat content and is often preferred in clinical trials to track changes in steatosis over time [13, 14]. Magnetic resonance elastography (MRE) further enhances the non‐invasive evaluation of fibrosis with superior diagnostic accuracy compared to FibroScan, although its availability is limited by cost and accessibility [11, 12]. In parallel, several serum‐based biomarkers and composite scoring systems have been developed to estimate fibrosis severity. These include the Fibrosis‐4 (FIB‐4) index and the NAFLD fibrosis score (NFS), which utilize common laboratory and demographic parameters to stratify patients by fibrosis risk [12, 13, 14]. More advanced panels, such as the Enhanced Liver Fibrosis (ELF) test and proprietary biomarker combinations like NIS4, offer promising performance in detecting advanced disease and distinguishing at‐risk MASH [12]. While these tools do not yet replace liver biopsy for definitive histological characterization, they serve as valuable, less invasive alternatives for patient evaluation and longitudinal monitoring in both clinical and research settings.

## 3 Current Therapies/Preventive Measures

The American and European societies for the study of the liver recommend diet, weight loss, and physical activity as the foundation of treatment for MASLD, formerly known as NAFLD. This approach emphasizes reducing overall energy intake, avoiding processed foods high in added fructose, and adopting a Mediterranean diet rich in monounsaturated fats, fiber, and antioxidants to improve liver health [6, 10]. Current guidelines advise engaging in moderate‐intensity aerobic exercise for 150–200 min per week, divided into three to five sessions, to support weight loss and metabolic improvements. Due to the limited availability of effective pharmacological therapies, vitamin E and the peroxisome proliferator‐activated receptor gamma (PPAR‐γ) agonist pioglitazone are sometimes recommended, particularly for non‐diabetic patients and those with biopsy‐proven NASH [10, 11]. However, these medications are associated with risks such as weight gain, fluid retention, and potential cancer concerns.

Black coffee has demonstrated significant benefits for liver health by reducing inflammation and hepatic steatosis and potentially aiding in fibrosis regression, especially in conditions like MASLD and cirrhosis [15]. These effects occur through caffeine‐dependent mechanisms, where caffeine blocks adenosine receptors (A1, A2A, A2B) to reduce inflammation and fibrogenesis and enhances lipid metabolism. Caffeine‐independent mechanisms involve bioactive compounds like cafestol, kahweol, and polyphenols, which provide antioxidant and anti‐inflammatory effects [15]. These compounds improve cholesterol metabolism via Cyp7b1 activation and regulate inflammatory signals by lowering CCL2 and increasing IL‐6, supporting liver regeneration [15].

In the absence of FDA‐approved medicines, lifestyle and dietary interventions remain the primary treatment strategy for MASLD (Tables 1 and 2) [16]. Emerging therapies like Resmetirom (MGL‐3196), a selective thyroid hormone receptor‐β (THR‐β) agonist, offer promising advancements by targeting liver fat metabolism.

**Table 1:** Current approaches to treating non‐alcoholic fatty liver disease (MASLD).

Modality	Effect
Diet:	
	‐ Weight reduction of 5%–10%: Moderate calorie restriction, reducing intake by 500–750 kcal/day. Enhances tissue structure in NASH. Prospective research indicates that consuming fructose poses a risk factor for MASLD [7].
	‐ Eliminate or significantly reduce saturated fats and fructose in the diet. Fructose boosts fat production by activating pyruvate dehydrogenase [7].
	‐ Consider omega‐3 supplementation: May reduce hepatic fat accumulation and triglyceride levels [7].
Physical activity:	
	‐ ≥ 250 min/week: Reduces insulin resistance and hepatic fat accumulation [7].
Pharmacological treatment:	
	‐ Vitamin E 800IU/day: Enhances tissue structure in NASH [7].
	‐ Pioglitazone 30 mg/day: Enhances tissue structure in NASH [7].
	‐ Metformin: Improves metabolic parameters and facilitates moderate weight loss. There is no direct improvement observed in MASLD. Its application is reserved for managing patients with fatty liver alongside Type 2 diabetes [7].
	‐ Statins: Limited evidence regarding tissue structure improvement. Safe for individuals with MASLD and reduces the likelihood of cardiovascular diseases [7].
Bariatric surgery:	
	‐ Roux‐en‐Y gastric bypass; adjustable gastric band; vertical gastrectomy: Enhances tissue structure in NASH in up to 80% cases, including fibrosis [7].

**Table 2:** Overview of existing therapies for MASLD.

Drug class	Mechanism of action	Strengths	Limitations
PPAR Agonists (e.g., Lanifibranor)	Increase lipid metabolism and insulin sensitivity via activating peroxisome proliferator‐activated receptors	Substantial decline in intrahepatic triglycerides (~50%); enhanced insulin sensitivity and adiponectin levels	Potential side effects; long‐term safety data limited
GLP‐1 Receptor Agonists (e.g., Semaglutide)	Increase insulin production, promote weight reduction, and have anti‐inflammatory properties	Significant weight loss, decreased liver fat, and improved liver histology overall	High expense, gastrointestinal side effects, and ongoing research on long‐term liver results
SGLT2 Inhibitors (e.g., Empagliflozin)	Lower weight and enhance glycemic management by blocking renal glucose reabsorption	Better glucose management, weight reduction, and cardiovascular advantages	Insufficient direct proof of fibrosis resolution; not authorized for MASLD explicitly
Vitamin E	Antioxidant that reduces oxidative stress in hepatocytes	Improvement in non‐diabetic individuals’ liver histology and enzymes	Long‐term safety issues (such as the risk of prostate cancer); no benefit in diabetic people
FXR Agonists (e.g., Obeticholic Acid)	Reduce hepatic lipogenesis and bile acid production by activating the farnesoid X receptor	Fibrosis improvement in certain clinical studies	Pruritus and dyslipidemia are among the side effects; safety issues prevent approval

## 4 Resmetirom as Drug Therapy

**Figure 1:**
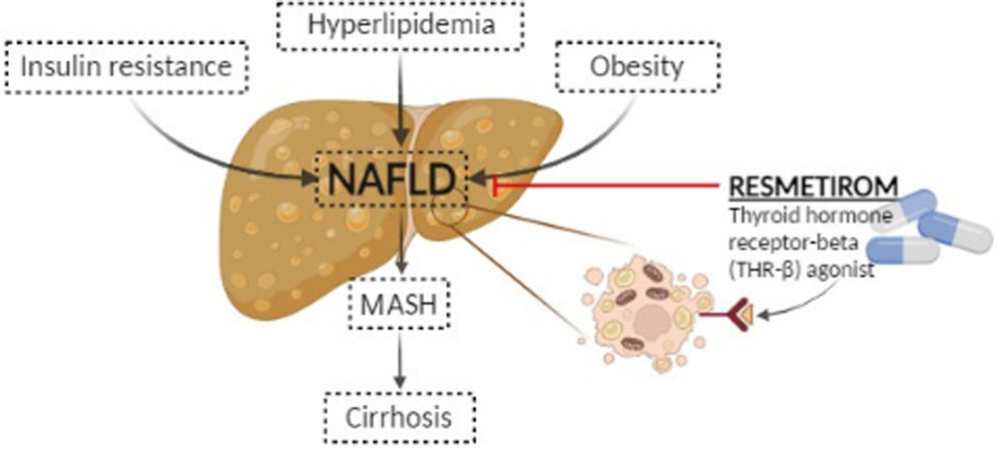
Resmetirom and its action on MASH.

Resmetirom, marketed as Rezdiffra, represents a promising and groundbreaking treatment approach for individuals diagnosed with noncirrhotic NASH accompanied by moderate to severe liver fibrosis, explicitly corresponding to stages F2–F3 fibrosis [17]. This treatment received approval from the FDA following Phase 3, randomized, placebo‐controlled trials [17]. Functioning as a THR‐β agonist, it is recommended as an adjunct to diet and exercise regimens. THR‐β is the predominant type of thyroid hormone receptor in the liver, and its activation by Resmetirom reduces intrahepatic triglycerides [17]. In contrast, THR‐α primarily governs thyroid hormone functions outside the liver, such as in the heart and bones [17]. Activation of hepatic THR‐β enhances mitochondrial biogenesis, increases fatty acid oxidation, and reduces hepatic lipotoxicity, leading to meaningful reductions in liver fat and improvement in metabolic parameters. In contrast, activation of THR‐α, which is primarily expressed in the heart, skeletal muscle, and central nervous system, has been associated with undesirable systemic effects, including tachycardia, bone loss, and neuropsychiatric symptoms [18, 19]. Earlier thyroid hormone analogs lacked isoform selectivity and were limited by such adverse outcomes. By acting specifically on THR‐β, Resmetirom mimics the beneficial hepatic effects of endogenous thyroid hormone while avoiding off‐target THR‐α stimulation, offering a more targeted, liver‐centric therapeutic strategy (Figure 1).

In an in vitro functional experiment assessing THR‐β activation, Resmetirom demonstrated 83.8% efficacy compared to triiodothyronine (T3), with an EC50 of 0.21 µM. Conversely, in the same functional assay for thyroid hormone receptor alpha (THR‐α) agonism, Resmetirom exhibited 48.6% efficacy compared to T3, with an EC50 of 3.74 µM [17].

The drug is generally well tolerated with minimal gastrointestinal side effects, including diarrhea, nausea, vomiting, and constipation [17]. Compared to the placebo group, there were slightly elevated incidences of gallstone‐related conditions such as cholelithiasis, acute cholecystitis, and obstructive pancreatitis among those receiving Resmetirom [17]. However, the overall occurrence rates for these events across all treatment groups were below 1 per 100 person‐years [17]. Resmetirom does not have any significant contraindications except for its avoidance in patients with decompensated cirrhosis [17]. The recommended dosage of Resmetirom varies based on the individual's body weight. For individuals weighing less than 100 kg, the advised dose is 80 mg orally once daily. The suggested dosage for those weighing 100 kg or more is 100 mg once daily with or without food [17].

## 5 Resmetirom's Efficacy

Resmetirom has undergone extensive clinical evaluation to assess its efficacy and safety in treating MASH. Two pivotal studies, the MGL‐3196‐05 and MAESTRO‐NAFLD‐1 trials, provide comprehensive insights into its clinical performance.

The MGL‐3196‐05 study, a 36‐week, double‐blind, placebo‐controlled trial, demonstrated that patients treated with Resmetirom exhibited a significant reduction in hepatic fat content by week 12 (−32.9%) and week 36 (−37.3%) compared to the placebo group (−10.4% and −8.5%, respectively) [20]. These results highlight Resmetirom's strong potential in effectively reducing liver fat accumulation, a crucial factor in managing MASH. Similarly, the MAESTRO‐NAFLD‐1 trial, a 52‐week randomized, double‐blind, placebo‐controlled phase 3 study, reinforced these findings [21]. Resmetirom significantly reduced atherogenic lipid levels, including LDL‐C, apo B, and triglycerides, particularly at the 100 mg dose [21]. Additionally, patients experienced improvements in hepatocyte injury markers, with substantial reductions in cytokeratin‐18 (CK‐18) fragments, underscoring Resmetirom's dual benefit for liver health and cardiovascular risk reduction [21].

Despite these promising outcomes, Resmetirom treatment was associated with a higher incidence of treatment‐emergent adverse events (TEAEs) compared to placebo [21]. Common adverse effects included gastrointestinal disturbances such as diarrhea, nausea, vomiting, and constipation. Moreover, patients receiving Resmetirom showed a slightly elevated occurrence of gallstone‐related complications, including cholelithiasis, acute cholecystitis, and obstructive pancreatitis, though the overall incidence remained low (< 1 per 100 person‐years) [21]. Although these events were not frequent overall, their occurrence suggests the need for clinical vigilance, particularly in patient subgroups predisposed to biliary complications. This includes individuals with a history of gallstones, rapid weight loss, or underlying gallbladder disease, as well as those receiving concomitant therapies known to influence biliary motility or lipid metabolism [22, 23].

The pooled analysis of three high‐quality randomized controlled trials involving 2231 participants demonstrated that Resmetirom significantly improves key clinical outcomes in MASH and liver fibrosis [23]. Treatment was associated with a marked reduction in hepatic fat fraction (SMD: −4.61, 95% CI: −6.77 to −2.44, *p* < 0.001), indicating strong lipid‐lowering activity within the liver. A greater proportion of patients achieved MASH resolution without fibrosis progression compared to placebo (RR: 2.51, 95% CI: 1.74–3.64, *p* < 0.001), highlighting its disease‐modifying potential. Additionally, improvements in fibrosis stage (RR: 2.31, 95% CI: 1.20–4.44, *p* = 0.01) support an antifibrotic effect. Beyond histological outcomes, Resmetirom also produced consistent enhancements in lipid profiles, liver enzyme levels, and MASH‐specific biomarkers, reflecting broader metabolic benefits [22, 23].

However, Resmetirom's long‐term safety and efficacy remain areas requiring further investigation. Current studies have not conclusively demonstrated its ability to reverse liver fibrosis or prevent the progression to cirrhosis and HCC. Additionally, Resmetirom is contraindicated in patients with decompensated cirrhosis, limiting its application in advanced stages of liver disease. These limitations emphasize the necessity for long‐term, large‐scale clinical trials to fully understand the drug's risk‐benefit profile and its impact on disease progression. Further studies should assess the long‐term effects of Resmetirom on cardiovascular outcomes, given their strong association with MASH. It is also important to include diverse populations, particularly individuals from different ethnic backgrounds and those with advanced fibrosis, to ensure broader applicability of the findings.

## 6 Conclusion

MASLD is a growing global health issue closely linked to obesity, diabetes, and metabolic syndrome. Although liver biopsy remains the standard diagnostic method, non‐invasive imaging and biomarkers now play a key role in diagnosis and disease monitoring. Lifestyle changes, such as improved diet and regular exercise, remain the primary treatment strategy due to limited medication options. Recently approved Resmetirom shows promising results in reducing liver fat and improving fibrosis markers in specific patient groups. However, more research is necessary to fully understand its long‐term safety, effectiveness, and potential to prevent severe disease outcomes. Future studies should combine medical therapies with lifestyle management to address MASLD and reduce related complications.

## Author Contributions

**Laiba Shakeel:** conceptualization, investigation, writing – original draft, project administration. **Ayesha Shaukat:** writing – review and editing, investigation, visualization, validation. **Aymar Akilimali:** writing – review and editing, investigation, validation.

## Ethics Statement

Ethics approval from IRB is not required.

## Conflicts of Interest

The authors declare no conflicts of interest.

## Transparency Statement

The lead author Aymar Akilimali affirms that this manuscript is an honest, accurate, and transparent account of the study being reported; that no important aspects of the study have been omitted; and that any discrepancies from the study as planned (and, if relevant, registered) have been explained.

## Data Availability

Data sharing is not applicable to this article as no new data were created or analyzed in this study.
